# Genome Editing for Improving Crop Nutrition

**DOI:** 10.3389/fgeed.2022.850104

**Published:** 2022-02-09

**Authors:** Ai Nagamine, Hiroshi Ezura

**Affiliations:** ^1^ Faculty of Life and Environmental Sciences, University of Tsukuba, Tsukuba, Japan; ^2^ Tsukuba Plant Innovation Research Center, University of Tsukuba, Tsukuba, Japan

**Keywords:** genome editing, nutritional improvement, crops, CRISPR/Cas9, TALEN, high GABA tomato, high oleic soybean oil

## Abstract

Genome editing technologies, including CRISPR/Cas9 and TALEN, are excellent genetic modification techniques and are being proven to be powerful tools not only in the field of basic science but also in the field of crop breeding. Recently, two genome-edited crops targeted for nutritional improvement, high GABA tomatoes and high oleic acid soybeans, have been released to the market. Nutritional improvement in cultivated crops has been a major target of conventional genetic modification technologies as well as classical breeding methods. Mutations created by genome editing are considered to be almost identical to spontaneous genetic mutations because the mutation inducer, the transformed foreign gene, can be completely eliminated from the final genome-edited hosts after causing the mutation. Therefore, genome-edited crops are expected to be relatively easy to supply to the market, unlike GMO crops. On the other hand, due to their technical feature, the main goal of current genome-edited crop creation is often the total or partial disruption of genes rather than gene delivery. Therefore, to obtain the desired trait using genome editing technology, in some cases, a different approach from that of genetic recombination technology may be required. In this mini-review, we will review several nutritional traits in crops that have been considered suitable targets for genome editing, including the two examples mentioned above, and discuss how genome editing technology can be an effective breeding technology for improving nutritional traits in crops.

## Introduction

The demand for food is increasing due to global population growth; the worldwide population is projected to require a 1.7-fold increase in cereals and a 1.8-fold increase in livestock products by 2050 (MAFF, Japan, 2019). Therefore, efforts should be made to increase food production using all possible methods. In addition, changing global dietary habits (from a starch-based diet to a high-fat, high-protein diet) and the aging of the population have led to an increase in lifestyle-related diseases, resulting in ever-increasing health concerns worldwide. Medical solutions are direct, but they are costly and not widely applicable. Therefore, it is important to address these lifestyle-related diseases with comprehensive nutritional intake through food. For these reasons, improving crop nutrition has become an important national policy in many countries. However, with conventional breeding techniques, it usually takes more than 10 years to produce a commercial cultivar that includes the desired traits. This is because many generations of crosses and backcrosses are generally required to accumulate a set of QTLs for the target traits. Some crop species have accumulated useful genetic resources that provide an important basis for creating new superior varieties with excellent genetic tools. However, it is difficult to overcome the time constraint, and time is one of the major hurdles in responding to ever-changing international needs.

The advent of genome editing technologies (ZFN, TALEN, CRISPR/Cas9, etc.) has attracted a great deal of attention because the remove the limitations of conventional breeding methods (for more information on each technology in crops, we recommend reading the following reviews: Rojas-Vásquez and Gatica-Arias, 2019; Matres et al., 2021). These technologies are capable of creating precise mutations in targeted genes, and their use in the field of crop breeding is on the rise. Some of the crops that are currently being produced include disease- and stress-tolerant crops, high oleic acid soybeans, and high GABA-accumulating tomatoes. If the advantages of genome editing are utilized to the fullest, it will be possible to modify the accumulation of target functional components while retaining 100% of the host’s other useful traits by controlling the expression and modifying the functions of genes involved in the pre- and postmetabolism of the target functional components. Therefore, genome editing technology is expected to be an effective breeding method to modify the metabolism of nutritional functional components, especially for crops whose biosynthetic systems are known.

In this mini-review, we discuss the current status of developmental research on the improvement of functional components in crops using genome editing technology and provide an outlook for the future.

## Improving Nutrient Composition by Controlling Gene Expression

One of the targets of genome editing is to regulate the expression level of genes involved in the metabolism of the target nutrient or undesirable substance itself. In this case, there are two targets: one is as far upstream of the CDS as possible, and the other is the untranslated region involved in regulating expression, including the 5′UTR. The former target induces a frameshift as far upstream as possible of the CDS and results in a termination codon that is not normally present, thereby achieving incomplete translation by premature termination of translation. The latter target aims to regulate the expression level of target genes and proteins by mutating the untranslated regions of genes involved in the regulation of transcription and expression levels.

### Development of High Oleic Soybean Oil

Soybean oil contains high amounts of polyunsaturated fatty acids such as linoleic acid and linolenic acid and low amounts of monounsaturated fatty acids such as oleic acid. Soybean oil is hydrogenated to improve its fatty acid composition, but this process produces trans-fatty acids that are rather harmful to human health (Ascherio et al., 2008). In 2019, Calyxst (Minnesota, United States) developed a new soybean with more than 80% oleic acid (similar to olive oil) using TALEN and launched Calyno^TM^, the world’s first genome-edited soybean with improved oleic acid content. They achieved the goal of more than 80% oleic acid and less than 3% linolenic acid by knocking out the fatty acid desaturase *FAD2-1A, FAD2-1B* and *FAD3A* exons, which metabolize oleic acid to linoleic acid, using TALEN (Haun et al., 2014; Demorest et al., 2016). This strategy involves knocking out genes involved in the downstream metabolism of oleic acid synthesis in the soybean fatty acid metabolic pathway that essentially leads to linolenic acid, thereby increasing the accumulation of the intermediate product oleic acid and inhibiting the downstream synthesis of linolenic acid and linoleic acid.

## Other Examples

Similar genome editing has been carried out in many crops and many genes, including knockout of the vacuolar invertase gene *VInv*, which produces reducing sugars responsible for acrylamide production in potatoes (Clasen et al., 2016), and knockout of the *PPO* gene in mushrooms (Waltz, 2016a) (Table 1). The strategy of targeting the CDS to induce frameshifting often results in a simple disruption of the target gene, and even if the structure and function of the protein encoded by the target gene are not clear, it is easy to obtain the desired phenotype. The ease of application is one of the reasons why this strategy has been used in many cases. On the other hand, the traits that can be altered by this strategy are limited to those that occur when the molecular biological events that the target gene is responsible for are restricted, such as the repression of the synthesis or degradation of specific substances, repression of the conversion to downstream metabolites, repression of the transport to specific compartments, and so on. However, for the nutritional improvement of crops, when the goal is to increase the synthesis and accumulation of the target product, sometimes the “suppressive” modification described above is not sufficient to meet market needs.

**TABLE 1:** Applications of CRISPR/Cas9 in major crop species to improve nutritional contents.

Common name		Phenotype	Target gene	Target region	GE result on target expression or activity	GE technique	Molecular function of the target gene	References
Rice		increased content of resistant starch	*SBEI and SBEIIb*	CDS	DOWN	CRISPR/Cas9	Regulate amylose contents	Sun et al. (2017)
Rice		low cesium accumulation	*OsHAK-1*	CDS	DOWN	CRISPR/Cas9	Cs + uptake in roots	Nieves-Cordones et al. (2017)
Sorghum		increased digestibility and protein quality	*k1C gene family*	n.i.	DOWN	CRISPR/Cas9	α-kafirins (major storage proteins)	Li et al. (2018a)
Bread wheat		low gluten content	*sgAlpha-1 sgAlpha-2*	CDS	DOWN	CRISPR/Cas9	the immunoreactive α-gliadin	Sánchez-León et al. (2018)
Soybean		altered fatty acids levels	*FAD2-1A* and *FAD2-1B*	CDS	DOWN	TALEN	Fatty acid desaturase 2	Haun et al. (2014); Demorest et al. (2016)
Peanut		increased oleic acid content	*FAD2A* and *FAD2B*	CDS	DOWN	CRISPR/Cas9	Converts oleic acid to linoleic acid gene coding sequences	Yuan et al. (2019)
Peanut		0.5–twofold increase in the oleic acid content	*FAD2*	CDS	DOWN	TALEN	Converts oleic acid to linoleic acid gene coding sequences	Wen et al. (2018)
Sweet potato		decreased amylose content	*GBSSI*	CDS	DOWN	CRISPR/Cas9	Granule-bound starch biosynthesis	Wang et al. (2019)
Sweet potato		decreased amylopectin content; increased amylose content	*SBEII*	CDS	DOWN	CRISPR/Cas9	Starch branching for amylopectin	Wang et al. (2019)
Potato		decreased browning	*PP02*	CDS	DOWN	CRISPR/Cas9	Converts phenolic substrates to quinones	González et al. (2020)
Potato		decreased steroidal glycoalkaloid content	*16DOX*	CDS	DOWN	CRISPR/Cas9	Steroidal glycoalkaloid biosynthesis	Nakayasu et al. (2018)
Potato		reduced levels of acrylamide	*Vinv*	CDS	DOWN	TALEN	Accumulation of reducing sugars which cause acrylamide accumulation.	Clasen et al. (2016)
Tomato		reduced concentration of γ-aminobutyric acid	*GABA-TP1, GABA-TP2, GABA-TP3, CAT9 and SSADH*	CDS	DOWN	CRISPR/Cas9	Essential genes for the γ-aminobutyric acid (GABA) pathway	Li et al. (2018b)
Tomato		Pink tomatoes	*MYB12*	CDS	DOWN	CRISPR/Cas9	Flavonoids Metabolic Pathways	Deng et al. (2018); Zhu et al. (2019)
Tomato		oprange tomatoes and yellow tomatoes, respectively	*CRTISO or PSY1*	CDS	DOWN	CRISPR/Cas9	Carotenoids Metabolic Pathways	Dahan-Meir et al. (2018)
Tomato		purple tomatoes	*SlANT1*	Promo-ter	UP	TALEN and CRISPR/Cas9	Anthocyanin biosynthesis	Čermák et al. (2015)
Tomato		5.1-fold increase in the lycopene content	*SGR1, LCY-E, Blc, LCY-B1, and LCY-B2*	CDS	DOWN	CRISPR/Cas9	Carotenoids Metabolic Pathways	Li et al. (2018d)
*	Tomato	increased carotenoid, lycopene, and β-carotene	*SlDDB1, SlDET1, SlCYC-B*	CDS	DOWN	Target-AID	Carotenoids Metabolic Pathways	Hunziker et al. (2020)
*	Tomato	sevenfold to 15-fold increase in GABA accumulation	*SlGAD2 and SlGAD3*	CDS (AID)	UP	CRISPR/Cas9	Aminobutiric acid Metabolic Pathways	Nonaka et al. (2017)
Wild tomato		increased vitamin C content	*GGPI*	uORF	UP	CRISPR/Cas9	Vitamin C metabolism	Li et al. (2018b)
Tomato		decreased anthocyanin content	*SlANT2, SlAN2-like*	CDS	DOWN	CRISPR/Cas9	Anthocyanin biosynthesis	Yan et al. (2020); Zhi et al. (2020)
Tomato		decreased anthocyanin content	*HYS*	CDS	DOWN	CRISPR/Cas9	Anthocyanin biosynthesis in response to light	Qiu et al. (2019)
Tomato		increased phenylalanine-derived volatile content	*FLORAL4*	CDS		CRISPR/Cas9	Regulates phenylalanine-derived volatiles in fruit	Tikunov et al. (2020)
Tomato		decreased volatile organic compounds	*RIN*	CDS	DOWN	CRISPR/Cas9	Ripening control via ethylene	Ito et al. (2017); Zhi et al. (2020)
Tomato		SSC, fiber, fructose, ascorbic acid, total phenol, carotene, oxalic acid	*L1L4*	CDS	DOWN	ZFN	Metabolite pathway	Gago et al. (2017)
Tomato/wild tomato		high lycopene content	*cycB*	CDS	DOWN	CRISPR/Cas9	Metabolite pathway	Zsögön et al. (2018)
Eggplant		decreased browning	*PP04, PPOS, and PP06*	CDS	DOWN	CRISPR/Cas9	Converts phenolic substrates to quinones	Maioli et al. (2020)
Grape		decreased tartaric acid content	*IdnDH*	CDS	DOWN	CRISPR/Cas9	Tartaric acid biosynthesis Vegetables	Ren et al. (2016)
Carrot		decreased anthocyanin content	*F3H*	CDS	DOWN	CRISPR/Cas9	Anthocyanin biosynthesis	Klimek-Chodacka et al. (2018)
*Brassica rapa*		decreased fructose, glucose, and increase sucrose contents	*BrOG1A and BrOG1B*	CDS	DOWN	CRISPR/Cas9	Primary metabolism	Jiang et al. (2020)
Rapeseed		increased seed oil content	*SFAR4 and SEARS*	CDS	DOWN	CRISPR/Cas9	Oil degradation	Karunarathna et al. (2020)
Rapeseed		increased oleic acid content; decreased linoleic and linolenic acid contents	*FAD2*	CDS	DOWN	CRISPR/Cas9	Fatty acid biosynthesis	Okuzaki et al. (2018)
Chinese kale		yellow color of Chinese kale with improved market prospects	*BoaCRTISO*	CDS	DOWN	CRISPR/Cas9	Carotenoid biosynthesis	Sun et al. (2020)
Lettuce		increased oxidation stress tolerance and ascorbate content	*LsGGP2*	uORF	UP	CRISPR/Cas9	Deleted uORFs of LsGGP2 to increase the translation of mRNAs	Zhang et al. (2018)
Banana		increased F-carotene content	*LCYe*	CDS	DOWN	CRISPR/Cas9	ß-carotene metabolism	Kaur et al. (2020)
Mush-room		decreased browning	*PPO*	CDS	DOWN	CRISPR/Cas9	Converts phenolic substrates to quinones	Waltz, (2016b), *review
Pome-granate		unique accumulation of gallic acid 3-0- and 4-0-glucosides	PgUGT84A23 and PgUGT84A24	CDS	DOWN	CRISPR/Cas9	UDP-dependent glycosyltransferases (UGTs) enzymes with overlapping activities in ß-glucogallin biosynthesis	Chang et al. (2019)

### Possibility of Regulating Expression by Improving the Untranslated Region

The transcriptional efficiency of genes is mainly controlled by the promoter region in the 5′UTR and the terminator region in the 3′UTR. In addition, there are transcriptional control regions called enhancers in the upstream and intergenic regions of genes. In the introns of some genes, there are also miRNAs that control or inhibit transcription. Furthermore, some transcribed mRNAs contain translation control regions (uORFs) that inhibit the translation of mRNAs, and in fact, there are some successful cases where vitamin C contents have been improved (Li et al., 2018c; Zhang et al., 2018). By targeting these elements and causing genomic mutations, it is possible to suppress or overexpress their expression levels without disrupting the CDS of the target gene. To date, there are few reports of genome editing targeting these elements, but the Supplementary Table S1 summarizes the most likely examples. Although not within the scope of this review because crop nutrition improvement was not the target, a study in rice demonstrated that precise multiple-base editing at miRNA target sites is possible (Ohtsuki et al., 2018).

## Improving Nutrient Composition by Regulating Gene Function

Another goal of genome editing is to regulate the functions of proteins encoded by genes involved in the metabolism of a nutrient of interest or an unfavorable substance. In this case, the target may be the active center of the enzyme, the binding region of a ligand, or the activity control domain. Various patterns are possible depending on the combination of the physiological and structural properties of the target protein and the target trait.

### Development of Tomatoes With High Accumulation of GABA

Tomatoes have been an excellent source of GABA among crops (Briguglio et al., 2018; Gramazio et al., 2020), and they are also a major crop commonly eaten around the world.

Sanatech Seed Co., Ltd. (Tsukuba, Japan) launched the first genome-edited tomato in Japan, “Sicilian Rouge High GABA”, in 2021. This high-GABA tomato contains approximately four to five times the amount of GABA found in ordinary tomatoes. Considering that the high GABA tomatoes (without genome editing technology) available on the market until now contained approximately 1.5 times more GABA, this increase in the GABA content is a revolutionary improvement. CRISPR/Cas9 genome editing, which is responsible for this increase, targets the autoinhibitory domain (AID) on the C-terminal side of *GAD3*, an enzyme involved in the biosynthesis of GABA (Nonaka et al., 2017). By inducing a frameshift in this autoinhibitory domain, early termination of translation occurred, and the autoinhibitory domain of *GAD3* was excised (Nonaka et al., 2017). This strategy increases the enzymatic activity per molecule involved in GABA biosynthesis by eliminating inhibitors of *GAD3*, whose activity is normally suppressed, without modifying the expression level of *GAD3* itself.

### Other Examples

As mentioned earlier, when modifying the function of a gene by genome editing, it is necessary to know the function of the protein or peptide encoded by the gene at the molecular level. The fact that there are far fewer examples of genome editing that have actually been implemented and have been effective compared to genome editing for knockout purposes (Table 1, asterisks) makes it easy to imagine the many challenges that need to be overcome. However, TARGET-AID has proven that it is possible to reproduce amino acid substitution mutations and obtain phenotypes by genome editing technology when the mutation is known (Hunziker et al., 2020).

### Understanding the Molecular Mechanism of Protein Function Regulation is Necessary for Controlling Gene Function by Genome Editing

In many cases, the normal function of a protein is achieved by a complex interplay of various factors, including the regulatory conditions of the active/inactive form. Therefore, the selection of targets for genome editing requires both molecular biological and biochemical knowledge of molecular mechanisms such as protein domain structure, protein–protein interactions, and activation control by feedback/feed-forward regulation.

On the other hand, genome editing can be useful for basic research to obtain such knowledge. Compared to conventional gene transfer methods (e.g., overexpression and RNA interference), genome editing has the advantage of removing the effects of foreign genes and finely modifying the targets at the domain and base levels, which is the same advantage that genome editing brings to crop breeding.

## Future Prospects and Challenges

**FIGURE 1:**
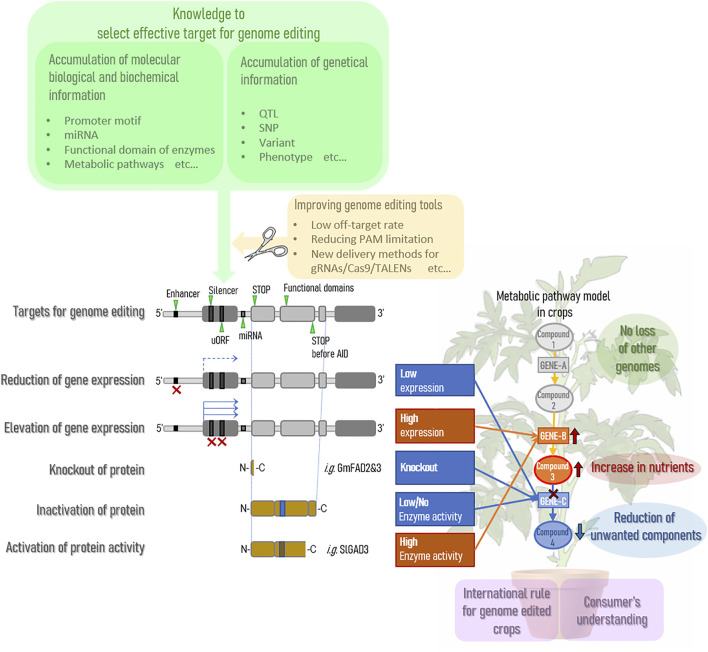
Current situation on gene editing for enhanced nutrition in crops.

Genome editing technology is expected to expand as a way to improve the nutritional status of agricultural crops. Currently, many QTLs have accumulated in crops on the market through breeding, and genome editing technology is expected to improve the nutritional status of crops without compromising almost 100% of these useful QTLs. In other words, it can dramatically improve the effort to maintain QTLs by backcrossing and greatly reduce the time and cost of new breeding endeavors. However, there are several points that need to be improved before stable practical applications can be achieved. A summary is provided in Figure 1.

### It is Necessary to Improve the Technology to Modify the Target Genes More Precisely

Current genome editing technologies cannot completely eliminate the risk of off-target effects. To overcome this weakness, various challenges are being addressed to improve the off-target rate (Manghwar et al., 2020), including the identification of factors affecting the occurrence of off-target effects (Modrzejewski et al., 2020) and the development of CRISPR/Cas type I-D (Osakabe et al., 2020). When specific motifs are already known in the promoter region, the need to substitute arbitrary bases is expected to increase and improvements in nickase and base editing technologies (Nishida et al., 2016; Sakata et al., 2019) will be increasingly required; these improvements will be accelerated as our understanding of the molecular mechanisms of key trait expression increases. In addition, when using the CRISPR/Cas9 system, a PAM sequence is currently required in the vicinity of the region of the target gene for which genome editing is desired; however, this is a major barrier to target selection. Currently, the challenge is to improve and eliminate this limitation of PAM sequences (Collias and Beisel, 2021).

### Understanding the Molecular Mechanism of Expression for Important Traits in Target Crops is Necessary

In the future, as the accuracy of the technology for modifying target genes improves, the effect of genome editing can be maximized by controlling point (SNP)-level mutations for efficient expression of traits. For this purpose, it is more important to understand the underlying molecular mechanisms. Thus, it will become increasingly important to collect and store diverse genetic resources and analyze them to accumulate more basic research knowledge on the target crop and more SNP information related to phenotypes.

### There is a Need to Establish Rules for Genome Editing Crops in Each Country

As we have discussed, since the discovery of the CRISPR/Cas9 mechanism in 2012, the progress of genome editing and its introduction into basic and applied science has accelerated worldwide. For the legality of this technology to persist, international rules must be generated quickly and appropriately.

Genome editing can be broadly classified into three categories depending on the type of mutation being introduced: SDN-1 uses nonhomologous end joining (NHEJ) to make relatively small deletions, insertions, and base substitutions; SDN-2 uses a species own homologous region as a template for homologous recombination repair (HDR); and SDN-3 introduces genes from outside of the species for repair. For each of these three types, there is currently an international debate on whether genome-edited crops should be treated as recombinant (GM) or nonrecombinant (non-GM). In fact, the treatment of genome-edited crops varies from country to country, with many European countries treating genome-edited crops in a more restrictive manner. In this context, in 2020, EFSA reported its view that the SDN-1 and SDN-2 types are not subject to risk assessment if they do not contain exogenous DNA (Naegeli et al., 2020).

In addition, on april 29, 2021, the European Commission published the results of its review of the place of “new genomic technologies (NGTs)" in EU law, which strongly suggests that there are limits to the ability of existing legislation to apply to NGTs and their products and that legislation needs to be adapted to scientific and technological advances (https://ec.europa.eu/food/plants/genetically-modified-organisms/new-techniques-biotechnology/ec-study-new-genomic-techniques_en). Furthermore, on 29 September 2021, a statement was issued by the UK government on its plans to lift GMO-like restrictions on genome editing (https://www.gov.uk/government/consultations/genetic-technologies-regulation/outcome/genetic-technologies-regulation-government-response).

While we should continue to monitor the views of other countries, the fact that the high oleic soybeans and high GABA tomatoes, which are categorized as SDN-1, have finally reached the market and are now available to the general public is a large step in the history of genome-edited crops and is certainly a major benchmark for countries to formulate future directions and appropriate rules.

### There is a Need to Improve Consumer Understanding of Genome-Edited Crops

Needless to say, the ultimate recipients of the developed genome-edited crops are consumers. When ordinary consumers purchase genome-edited crops at supermarkets, they are most likely to be concerned about safety. For consumers to understand the safety of genome-edited crops, it is necessary to communicate as correctly and clearly as possible how genome-edited crops were developed, why they are safe, what makes them different from conventional crops, and what makes them different from GM crops. Sanatech Seed Co., Ltd., the company that developed the high GABA tomato, has established two websites, one in Japanese and one in English, with Q&A pages for general questions, thus creating a platform of information that ordinary consumers can refer to when they are curious (https://sanatech-seed.com/en/). Web tools such as social networking services (SNSs), which are currently undergoing remarkable technological innovation, can be used as tools that provide opportunities for multidirectional communication, unlike traditional one-way mass media. Therefore, by using these new tools, we may be able to accomplish the task of information dissemination more effectively. On the other hand, until the new products created by this new technology are widely accepted by the public, consumers may be need to select and examine more appropriate information without getting caught up in sensational topics. Scientists and the international community should remain equally (or even more) loyal to the consumer’s motivation to understand these technologies.

## Discussion

Just 8 years after the publication of the first paper on CRISPR/Cas9 by Charpentier and Doudna in 2012 (Jinek et al., 2012), the Nobel Prize in Chemistry was awarded to CRISPR/Cas9 in 2020. The launch of the first CRISPR/Cas9 genome-edited crop in 2021 is a testament to the superiority of CRISPR/Cas9 as a crop breeding technology and reflects the current pressure on the breeding field and the international community to solve food supply problems. This Nobel Prize-winning genome editing technology is anticipated to help improve global nutrition.
