# Supplementary material for: Genome Editing for Improving Crop Nutrition
Source: Front Genome Ed. 2022 Feb 9;4:850104. doi: 10.3389/fgeed.2022.850104 (PMC8864126; doi:10.3389/fgeed.2022.850104)
Supplement: Supplementary file 1 [file Table1.DOCX]

Supplementary Material

**Supplementary Table - Possibility of regulating expression by improving the untranslated region.**

This is an example of an expression control element in an untranslated region that has not yet been genome-edited, but could be a promising target for genome editing to improve the expression of specific genes in the future.
